# Behavioral Activation as a Mechanism of Change in Psychotherapy for Late-Life Depression

**DOI:** 10.1093/geroni/igaf122.3046

**Published:** 2025-12-31

**Authors:** Lilla Brody, Maayra Butt, Brenna Renn

**Affiliations:** University of Nevada, Las Vegas, Henderson, Nevada, United States; University of Nevada, Las Vegas, Las Vegas, Nevada, United States; University of Nevada, Las Vegas, Las Vegas, Nevada, United States

## Abstract

Late-life depression is a common condition that, left untreated, exacerbates negative health outcomes. Despite availability of effective psychotherapeutic treatments for depression, few trials have explicitly investigated treatment mechanisms. Engage is a new, brief behavioral psychotherapy for older adults with depression. This secondary data analysis aims to evaluate behavioral activation as a mediator of the relationship between baseline and post-test depression, hypothesizing that behavioral activation would be a mechanism of change for those in the Engage group. Analysis included 92 older adults with depression who participated in the randomized controlled noninferiority trial comparing Engage with Problem Solving Therapy (PST). Depression (measured via the Hamilton Depression Rating Scale; HAM-D) and behavioral activation (measured via the Behavioral Activation for Depression Scale; BADS) were recorded at baseline and 9 week follow up. Participants were an average of 67.5 years old (SD = 6.19), mostly female (70.7%), Non-Hispanic (92.4%), and white (85.9%) . Analyses were conducted in R and utilized the bruceR package for mediation. HAM-D scores improved for both groups (ps < 0.05). Separate mediation analyses in the Engage and PST groups indicated a direct effect between HAM-D scores across time (p < 0.01). Change in BADS was associated with significant decreases in HAM-D across time for both groups. Results suggest that behavioral activation contributes to but does not fully explain change in depressive symptoms during brief psychotherapy for late-life depression. Psychotherapy treatment mechanisms warrant further study in more diverse samples to better understand treatment response.

